# N-Terminal Tagging with GFP Enhances Selectivity of Agitoxin 2 to Kv1.3-Channel Binding Site

**DOI:** 10.3390/toxins12120802

**Published:** 2020-12-16

**Authors:** Oksana V. Nekrasova, Alexandra L. Primak, Anastasia A. Ignatova, Valery N. Novoseletsky, Olga V. Geras’kina, Ksenia S. Kudryashova, Sergey A. Yakimov, Mikhail P. Kirpichnikov, Alexander S. Arseniev, Alexey V. Feofanov

**Affiliations:** 1 Shemyakin-Ovchinnikov Institute of Bioorganic Chemistry, Russian Academy of Sciences, ul. Miklukho-Maklaya 16/10, 117997 Moscow, Russia; primak.msu@mail.ru (A.L.P.); aignatova_83@mail.ru (A.A.I.); rekamoskva@mail.ru (K.S.K.); sa-yakimov@yandex.ru (S.A.Y.); kirpichnikov@inbox.ru (M.P.K.); aars@nmr.ru (A.S.A.); avfeofanov@yandex.ru (A.V.F.); 2 Biological Faculty, Lomonosov Moscow State University, Leninskie Gory 1, 119992 Moscow, Russia; valery.novoseletsky@yandex.ru (V.N.N.); olgsamsonova@yandex.ru (O.V.G.)

**Keywords:** agitoxin, GFP, potassium channel, selective ligand

## Abstract

Recently developed fluorescent protein-scorpion toxin chimeras (FP-Tx) show blocking activities for potassium voltage-gated channels of Kv1 family and retain almost fully pharmacological profiles of the parental peptide toxins (Kuzmenkov et al., Sci Rep. 2016, 6, 33314). Here we report on N-terminally green fluorescent protein (GFP)-tagged agitoxin 2 (GFP-L2-AgTx2) with high affinity and selectivity for the binding site of Kv1.3 channel involved in the pathogenesis of various (primarily of autoimmune origin) diseases. The basis for this selectivity relates to N-terminal location of GFP, since transposition of GFP to the C-terminus of AgTx2 recovered specific interactions with the Kv1.1 and Kv1.6 binding sites. Competitive binding experiments revealed that the binding site of GFP-L2-AgTx2 overlaps that of charybdotoxin, kaliotoxin 1, and agitoxin 2, the known Kv1.3-channel pore blockers. GFP-L2-AgTx2 was demonstrated to be applicable as a fluorescent probe to search for Kv1.3 pore blockers among individual compounds and in complex mixtures, to measure blocker affinities, and to visualize Kv1.3 distribution at the plasma membrane of Kv1.3-expressing HEK293 cells. Our studies show that definite combinations of fluorescent proteins and peptide blockers can result in considerable modulation of the natural blocker-channel binding profile yielding selective fluorescent ligands of certain channels.

## 1. Introduction

Many of peptide toxins from animal venoms are highly specific ligands of potassium, sodium, calcium and acid-sensing channels, nicotinic acetylcholine, and NMDA receptors [1]. They are considered to be prospective drugs for the treatment of channel-associated diseases [2,3,4,5] and are widely used to study ion channel structure and function [6,7]. An ever-widening range of peptide ligand applications necessitate the production of fluorescently labeled ligands to make these applications more efficient and reliable. Diversity of fluorescently labeled ligands of ion channels and their applications were recently reviewed in [8]. 

Several fluorescently labeled peptide toxins were produced by chemical synthesis using reactive fluorescent organic dyes [9,10,11]. The procedures are usually laborious, and the yields of the labeled products are often low. As an alternative, genetically encoded fluorescent protein-scorpion toxin chimeras (FP-Tx), which are the polypeptide ligands of potassium voltage-gated Kv1-channels were recently developed [12]. These FP-Tx carry a fluorescent protein moiety at the N-terminus of the peptide ligand and exhibit high affinity to the target channels. Currently, the new type of fluorescent ligands is represented by two structurally different chimeras, namely green fluorescent protein (GFP) fused with peptide OSK1 (GFP-OSK1) and red fluorescent protein TagRFP fused with agitoxin 2 (RFP-AgTx2). Both chimeras retain the binding profiles and the pore blocking activities of corresponding toxins (OSK1 and AgTx2) for the channels Kv1.x (x = 1, 3, 6). At the same time, introduced voluminous tags interfere to some extent with the binding characteristics of toxin moieties decreasing affinity of RFP-AgTx2 (moderately) and GFP-OSK1 (drastically) toward Kv1.2 channel [12]. 

AgTx2 is a well-characterized peptide blocker of Kv1 channels with sub-nanomolar affinity to Kv1.1, Kv1.3, and Kv1.6 channels [13], and nanomolar affinity to Kv1.2 [14]. AgTx2 was successfully used in the form of a fluorescently labeled peptide (either RFP-AgTx2, or AgTx2 chemically labeled with tetramethylrhodamine, R-AgTx2) to probe binding affinities of various peptide toxins to different Kv1 channels [12,15,16,17,18]. Extending color variety of AgTx2-based FP-Tx, we report here on the design of GFP-tagged AgTx2. Ability of GFP-tagged AgTx2 to interact with the pore blocker binding site of Kv1.x (x = 1, 3, 6) was studied using bioengineering analytical systems on the basis of hybrid channels KcsA-Kv1.x (x = 1, 3, 6), which retain binding affinity and selectivity profiles of the eukaryotic Kv1 channels to small organic and peptide pore blockers [15,16,17]. 

We found a drastic increase in the selectivity of N-terminally GFP-tagged AgTx2 to Kv1.3 binding site over non-labeled AgTx2, as well as over labeled derivatives, R-AgTx2 and RFP-AgTx2, which were studied by us previously [11,12]. To study the basis of the unusual binding selectivity, modified structural analogs of GFP-tagged AgTx2 were constructed. It was shown that permutation of GFP from N- to C- terminus of AgTx2 recover high affinity of GFP-tagged AgTx2 to the ligand-binding sites of Kv1.1 and Kv1.6 channels in addition to that of Kv1.3. 

## 2. Results

### 2.1. Design and Properties of GFP-L2-AgTx2

**Figure 1:**
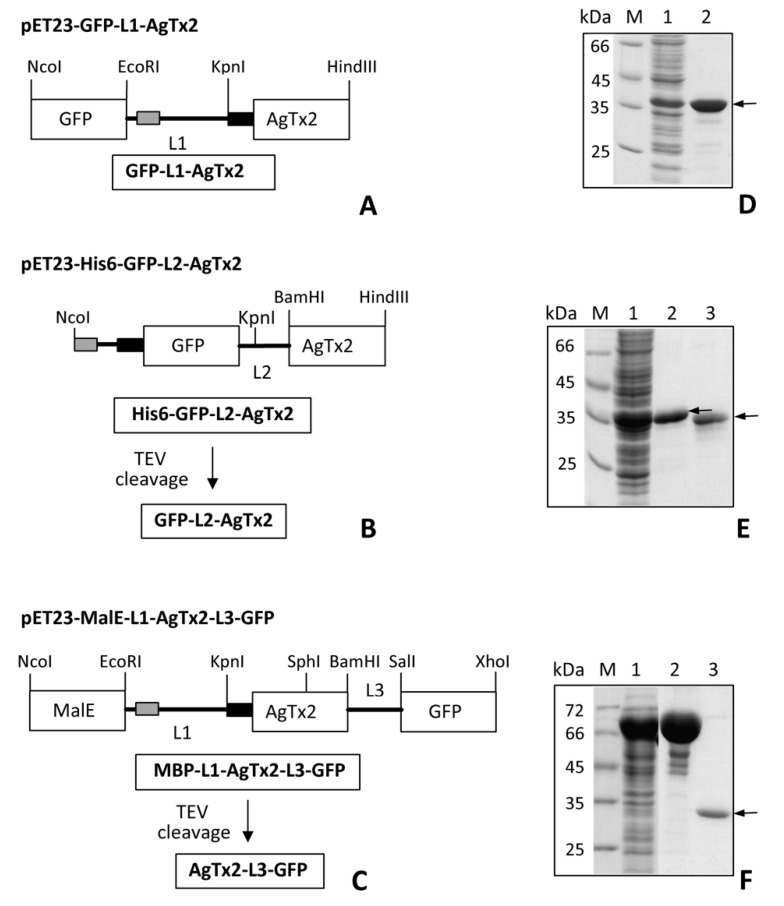
GFP-tagged AgTx2 proteins and their production in *E. coli*. (**A**–**C**). Schemes of GFP-tagged AgTx2 proteins and corresponding expression cassettes cloned in pET-23d vector are shown: GFP-L1-AgTx2 (**A**); His6-GFP-L2-AgTx2 and GFP-L2-AgTx2 (**B**); MBP-L1-AgTx2-L3-GFP and AgTx2-L3-GFP (**C**). GFP-L2-AgTx2 and AgTx2-L3-GFP were produced by TEV protease cleavage of His6-GFP-L2-AgTx2 and MBP-L1-AgTx2-L3-GFP, respectively. TEV protease cleavage site (ENLYFQG) is shown as a black rectangle. His6-tag is shown as a gray rectangle. MalE is a gene, encoding maltose binding protein (MBP). L1, L2, L3 are polypeptide linkers (see Section 5.1 for sequences). NcoI, EcoRI, KpnI, SphI, BamHI, SalI, XhoI and HindIII are cleavage sites of restriction endonucleases. (**D**–**F**). Coomassie-stained 12.0% SDS-PAGE gels show levels of total GFP-tagged AgTx2 biosynthesis in *E. coli* BL21(DE3) cells (lane 1 on each panel) and purified GFP-tagged AgTx2 proteins, namely GFP-L1-AgTx2 ((**D**), lane 2), His6-GFP-L2-AgTx2 and GFP-L2-AgTx2 ((**E**), lanes 2 and 3, respectively), MBP-L1-AgTx2-L3-GFP and AgTx2-L3-GFP ((**F**), lanes 2 and 3, respectively). M is a protein mass marker. Positions of the target proteins are shown by arrows.

In the designed chimeric protein GFP-L2-AgTx2 (Figure 1B), a fluorescent tag, GFP, is located at the N-terminus of the chimera and is separated from AgTx2 by a short flexible glycine-serine linker L2. The sequence of L2 is presented in Section 5.1. To produce GFP-L2-AgTx2, recombinant protein His6-GFP-L2-AgTx2 (Figure 1B) was expressed in *E. coli* cells and isolated from biomass by Ni-affinity chromatography. His6-tag was removed from His6-GFP-L2-AgTx2 by hydrolysis with Tobacco Etch Virus (TEV) protease at the TEV protease cleavage site placed after the His6-tag (Figure 1B). 

Interactions of GFP-L2-AgTx2 with the pore blocker binding sites of the Kv1.x (x = 1, 3, 6) channels were studied with the bioengineering systems based on *E. coli* spheroplasts that expose hybrid potassium channels KcsA-Kv1.x (x = 1, 3, 6) in their inner membrane. KcsA-Kv1.x (x = 1, 3, 6) hybrid channels bear extracellular binding sites of the corresponding eukaryotic Kv1 channels and provide both recognition of Kv1 channels pore blockers and evaluation of their affinity [15,16,17,18,19]. As systematically verified, data obtained with KcsA-Kv1.x (x = 1, 3, 6) for small organic compounds, peptide toxins, as well as fluorescent protein-tagged pore blockers are in agreement with the results of electrophysiological studies [12,16].

**Figure 2:**
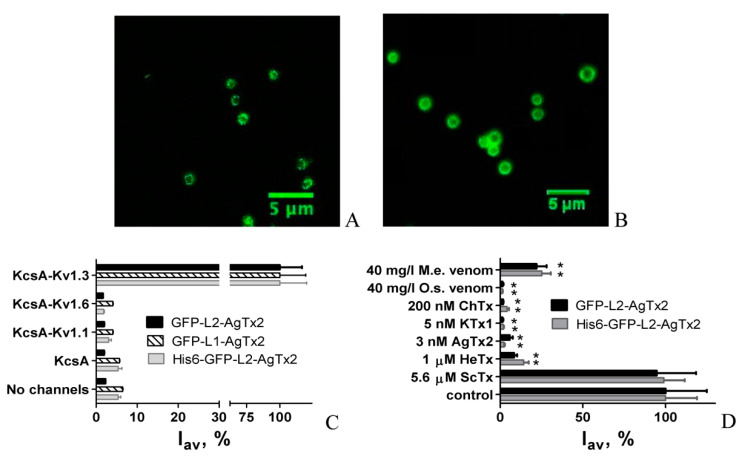
Interactions of GFP-tagged AgTx2 with spheroplasts presenting KcsA-Kv1.x (x = 1, 3, 6) and KcsA channels. (**A**,**B**) Typical confocal fluorescent image of GFP-L2-AgTx2 (20 nM) (**A**) and His6-GFP-L2-AgTx2 (20 nM) (**B**) bound to KcsA-Kv1.3 at the surface of spheroplasts are shown. (**C**) Binding of GFP-tagged AgTx2 chimeras (40 nM) to KcsA-Kv1.x- and KcsA-presenting spheroplasts and spheroplasts prepared from non-transformed *E. coli* cells are shown. (**D**) Comparison of the ability of peptide ligands (ChTx, KTx1, AgTx2, HeTx, and ScTx) as well as venoms of scorpions *M. eupeus* (M.e.) and *O. scrobiculosus* (O.s.) to competitively displace GFP-L2-AgTx2 (20 nM) and His6-GFP-L2-AgTx2 (20 nM) from KcsA-Kv1.3 embedded in the spheroplast membrane is presented. Control—binding of GFP-tagged AgTx2 (20 nM) to KcsA-Kv1.3 in the absence of competitors. I_av_ is the average fluorescence intensity of GFP-tagged AgTx2 associated with spheroplasts. I_av_ values are means of three independent experiments (mean ± SEM, *n* = 3). Asterisks mark values that are significantly (*p* < 0.05, unpaired two-tailed *t*-test) different from the corresponding control I_av_ value.

Our studies revealed that GFP-L2-AgTx2 binds readily to KcsA-Kv1.3-presenting spheroplasts (Figure 2A), while its binding to KcsA-Kv1.1- and KcsA-Kv1.6-presenting spheroplasts is very low and comparable to the level of weak nonspecific binding to KcsA-presenting spheroplasts or spheroplasts prepared from non-transformed *E. coli* cells (Figure 2C). This feature was preserved at different pH values ranging from 6.5 to 8.0 (not shown). 

**Figure 3:**
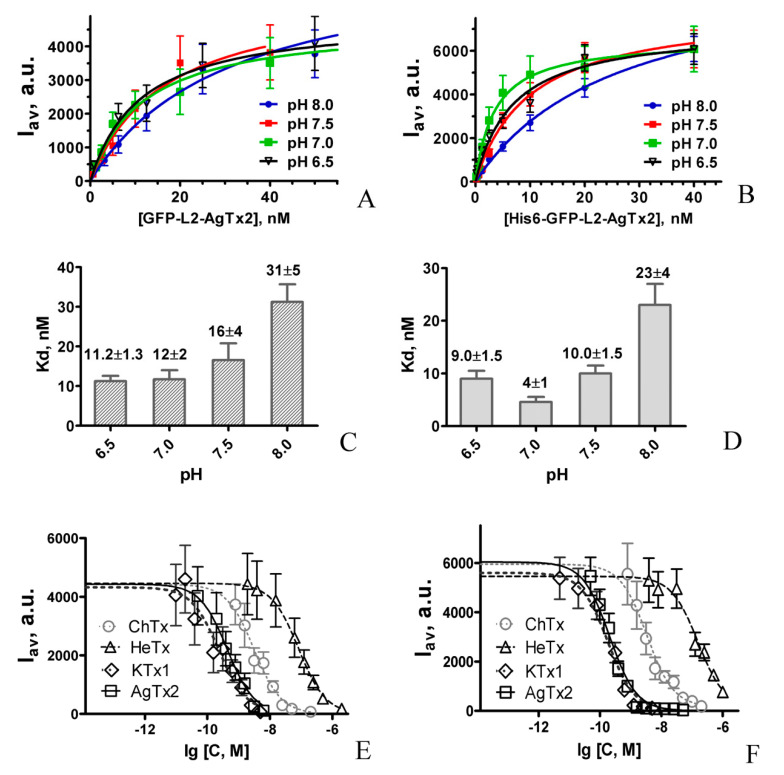
Concentration dependencies of KcsA-Kv1.3 binding to GFP-L2-AgTx2, His6-GFP-L2-AgTx2 and peptide blockers. (**A**,**B**). Concentration dependencies of GFP-L2-AgTx2 (**A**) and His6-GFP-L2-AgTx2 (**B**) binding to KcsA-Kv1.3 at different pH are shown. (**C**,**D**). pH-dependencies of the dissociation constants (*K_d_*) of KcsA-Kv1.3 complexes with GFP-L2-AgTx2 (**C**) or His6-GFP-L2-AgTx2 (**D**) are shown. (**E**,**F**). Competition between peptide blockers (ChTx, HeTx, KTx1, AgTx2; variable concentration C) and GFP-L2-AgTx2 (20 nM) (**E**) or His6-GFP-L2-AgTx2 (20 nM) (**F**) for the binding to KcsA-Kv1.3 is shown. I_av_ is the average fluorescence intensity of GFP-tagged AgTx2 associated with spheroplasts.

To verify specificity of GFP-L2-AgTx2 interaction with the Kv1.3 binding site on the surface of KcsA-Kv1.3-presenting spheroplasts, we have studied the effect of well-known potassium channel peptide blockers on this interaction (Figure 2D). High affinity blockers such as AgTx2, kaliotoxin 1 (KTx1) and charybdotoxin (ChTx) displaced GFP-L2-AgTx2 from the complexes with KcsA-Kv1.3 at the low nanomolar (AgTx2 and KTx1) and submicromolar concentrations (ChTx). Hetlaxin (HeTx), a moderate-affinity ligand of Kv1.3 channels [18], competed with GFP-L2-AgTx2 at low micromolar concentrations. Scillatoxin (ScTx), a pore blocker of KCa2.3 channel, which has no ability to interact with Kv1.3 channel, did not affect GFP-L2-AgTx2 binding to KcsA-Kv1.3. These results correlate with known relative affinities of the tested peptide blockers to Kv1.3 channel and suggest that GFP-L2-AgTx2 interacts with the same Kv1.3 binding site as the peptide blockers AgTx2, KTx1, ChTx, and HeTx. According to data presented in Figure 2C, GFP-L2-AgTx2 forms complexes with the Kv1.3 binding site selectively in contrast to AgTx2 that interacts with either of Kv1.x (x = 1, 3, 6) channels [13]. In our measurements, formation of GFP-L2-AgTx2 complexes with the Kv1.3 binding site was distinctly observed at the 1.25 nM concentration of the ligand (Figure 3A), whereas the complexes with the Kv1.1 and Kv1.6 binding sites were not recognized at the GFP-L2-AgTx2 concentration up to 250 nM. It means that the GFP-L2-AgTx2 has >200-fold selectivity for Kv1.3 over Kv1.1 and Kv1.6 binding sites. For comparison, AgTx2 has *ca*. tenfold selectivity for Kv1.3 over Kv1.1 and Kv1.6 channels [13].

Affinity of GFP-L2-AgTx2 to the Kv1.3 binding site was characterized quantitatively by measuring concentration dependencies of GFP-L2-AgTx2 binding to KcsA-Kv1.3 at different pH (Figure 3A) and determining dissociation constants (*K_d_*) of the formed complexes using equation 1. *K_d_* values were found to be pH-dependent: smaller at neutral and slightly acidic pH and higher at basic pH (Figure 3C). According to these data the most efficient interactions of GFP-L2-AgTx2 with the Kv1.3 binding site occurred in the 6.5–7.5 pH range, while brightness of the fluorescent probe estimated at the saturation of binding was stable in the 6.5–8.0 pH range. 

### 2.2. Study of Structural Factors Defining GFP-L2-AgTx2 Selectivity 

To clarify the structural basis of the advanced selectivity of GFP-L2-AgTx2, a homologous GFP-L1-AgTx2 ligand was produced, in which the 45-aminoacid L1 linker was used instead of the L2 linker (Figure 1A). Linker L1 was previously employed in RFP-L1-AgTx2 chimera, which displayed high affinities to three channels: Kv1.1, Kv1.3, and Kv1.6 [12]. GFP-L1-AgTx2 was found to interact selectively with KcsA-Kv1.3 channel just as the GFP-L2-AgTx2 chimera (Figure 2C), and its selectivity did not depend on pH changes from 6.5 to 8.0 (not shown). These results suggest that the linker structure is not the cause of the observed selectivity of GFP-tagged AgTx2. 

Since the structural difference between RFP-L1-AgTx2 and GFP-L1-AgTx2 lies in the type of fluorescent protein, one can conclude that the observed selectivity of GFP-L1-AgTx2 (as well as GFP-L2-AgTx2) originates from GFP, which modulates the binding profile of AgTx2. 

**Figure 4:**
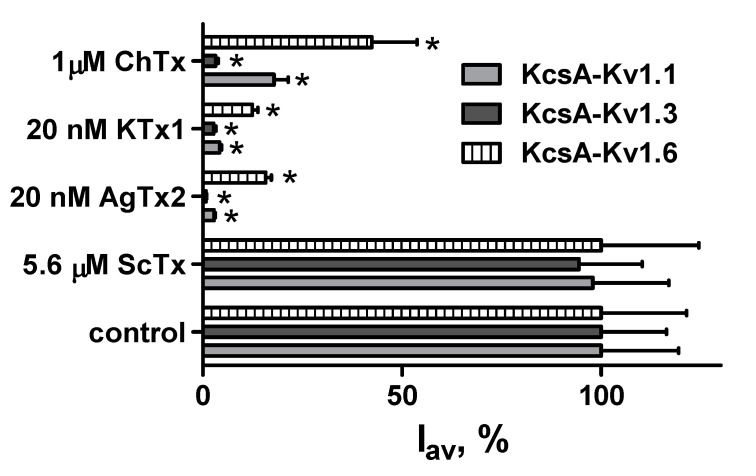
Interactions of AgTx2-L3-GFP with spheroplasts that express KcsA-Kv1.x (x = 1, 3, 6) channels. Binding of AgTx2-L3-GFP (20 nM) to KcsA-Kv1.x-presenting spheroplasts (control) and competitive displacement of AgTx2-L3-GFP from the complexes with KcsA-Kv1.x by peptide ligands (ChTx, KTx1, AgTx2 and ScTx) are shown. I_av_ is the average fluorescence intensity of AgTx2-L3-GFP associated with spheroplasts. I_av_ values are means of three independent experiments (mean ± SEM, *n* = 3). Asterisks mark values that are significantly (*p* < 0.05, unpaired two-tailed *t*-test) different from the control I_av_ value.

To evaluate the effect of shuffling the modules, C-terminally tagged AgTx2, namely AgTx2-L3-GFP, was constructed (Figure 1C). Fusion of AgTx2 with GFP was performed using the 20-aa glycine-rich L3 linker, whose sequence was similar to the L2 linker. In contrast to N-terminally tagged AgTx2, AgTx2-L3-GFP was found to interact with KcsA-Kv1.1 and KcsA-Kv1.6, as well as with KcsA-Kv1.3 (Figure 4). AgTx2-L3-GFP was readily displaced from the complexes with KcsA-Kv1.x (x = 1, 3, 6) by Kv1.x channel pore blockers AgTx2, KTx and ChTx. Relatively low ability of ChTx to compete with AgTx2-L3-GFP for the binding to KcsA-Kv1.x (x = 1, 6) is consistent with its lower affinity to Kv1.x (x = 1, 6), as compared to AgTx2 [13]. ScTx, taken as a negative control (it does not interact with Kv1.x (x = 1, 3, 6)), did not compete with AgTx2-L3-GFP for binding to KcsA-Kv1.x (x = 1, 3, 6) channels. Therefore, selectivity of GFP-tagged AgTx2 depends on the position of GFP in the construction: it is high for N-terminal and low for C-terminal fusion with AgTx2. 

### 2.3. Properties of His6-GFP-L2-AgTx2 Ligand

As described above, GFP-L2-AgTx2 was obtained from His6-GFP-L2-AgTx2 by cleavage with TEV protease (Figure 1B). We wondered whether it was necessary to delete His6-tag to achieve selective binding to the Kv1.3 binding site, especially, if GFP-L1-AgTx2 that contained His6-tag in the L1 linker demonstrated such selectivity (Figure 2C). 

Accordingly, we have evaluated interactions of His6-GFP-L2-AgTx2 with KcsA-Kv1.x (x = 1, 3, 6) and found that this FP-Tx preserved the binding profile (i.e., selectivity) of GFP-L2-AgTx2 at pH 7.5 (Figure 2C), as well as at other pH values in the 6.5–8.0 range (not shown). The observed interaction between His6-GFP-L2-AgTx2 and KcsA-Kv1.3 was specific as followed from competition between His6-GFP-L2-AgTx2 and Kv1.3 peptide blockers for the complex formation with the Kv1.3-binding site (Figure 2D). Complexes of His6-GFP-L2-AgTx2 with KcsA-Kv1.3 on the surface of spheroplasts were reliably detected at the ligand concentration of 1.25 nM (Figure 3B). At the same conditions, formation of His6-GFP-L2-AgTx2 complexes with KcsA-Kv1.1 and KcsA-Kv1.6 was not observed even after addition of 250 nM His6-GFP-L2-AgTx2. Hence, His6-GFP-L2-AgTx2 similar to GFP-L2-AgTx2 has >200-fold selectivity for Kv1.3 over Kv1.1 and Kv1.6 binding sites. 

Measurements of the dissociation constants for the complexes formed by His6-GFP-L2-AgTx2 revealed that *K_d_* value is smallest at neutral pH and increases *ca.* twofold at pH 6.5 and 7.5 and *ca*. sixfold at pH 8.0 (Figure 3B,D). His6-GFP-L2-AgTx2 demonstrates higher affinity to the Kv1.3-binding site and slightly modulated pH-profile as compared to GFP-L2-AgTx2 in the studied pH range. These data justified further study of His6-GFP-L2-AgTx2 to clarify an influence of His6-tag on the properties of FP-Tx in details, as well as to characterize this ligand as an alternative to GFP-L2-AgTx2. 

### 2.4. GFP-Tagged AgTx2 as a Component of the KcsA-Kv1.3-Based Bioengineering System

To characterize potential of GFP-L2-AgTx2 and His6-GFP-L2-AgTx2 as a component of the KcsA-Kv1.3-based bioengineering system for searching Kv1.3 channel ligands in complex mixtures, the response of complexes between KcsA-Kv1.3 and GFP-L2-AgTx2 or His6-GFP-L2-AgTx2 to addition of scorpion venoms was measured (Figure 2D). Venoms of scorpions *M. eupeus* and *O. scrobiculosus,* which contained peptide blockers of Kv1.3 channel, competitively displaced GFP-L2-AgTx2 and His6-GFP-L2-AgTx2 from the complexes with KcsA-Kv1.3 on the spheroplast membrane. As demonstrated above, only Kv1.3 blockers were able to interfere with complexation between KcsA-Kv1.3 and GFP-L2-AgTx2 or His6-GFP-L2-AgTx2. Therefore, both GFP-tagged chimeras in combination with KcsA-Kv1.3–bearing spheroplasts are suitable for recognition of Kv1.3 blockers among individual compounds and in multicomponent mixtures. This property seems to be especially useful, as it allows one to apply GFP-tagged AgTx2 for the analysis of crude venoms and their fractions at the stages of venom separation, as well as for the identification of individual channel blockers [16].

To verify applicability of GFP-L2-AgTx2 and His6-GFP-L2-AgTx2 (in combination with KcsA-Kv1.3–bearing spheroplasts) for evaluation of peptide blocker affinities to Kv1.3-binding site, detailed competitive binding measurements were performed with the Kv1.3 peptide blockers ChTx, HeTx, KTx1 and AgTx2 (Figure 3E,F). 

Closely related blockers, AgTx2 and KTx1, competed with GFP-L2-AgTx2 and His6-GFP-L2-AgTx2 for the binding to KcsA-Kv1.3 in the nanomolar concentration range, while higher concentrations of ChTx and, especially, HeTx were required to achieve the effect. The apparent dissociation constants K_ap_ calculated from the titration curves (Figure 3E,F) using Equation (2) are presented in Table 1 and demonstrate good similarity for two sets of experiments performed with different fluorescent ligands. Moreover, the calculated K_ap_ values (Table 1) are consistent with those reported previously for corresponding peptide blockers, when the competitive binding experiments were performed with R-AgTx2, RFP-L1-AgTx2 or GFP-OSK1 [12,15,18]. These results indicate that both GFP-L2-AgTx2 and His6-GFP-L2-AgTx2 can be successfully used as a component of the KcsA-Kv1.3-based bioengineering system to estimate affinities of different peptide blockers to Kv1.3 binding site. 

### 2.5. GFP-Tagged AgTx2 as a Fluorescent Probe of Kv1.3 on the Membrane of HEK293 Cells 

In the following experiments we have verified, if AgTx2-L2-GFP, His6-AgTx2-L2-GFP and GFP-L1-AgTx2 can be used as fluorescent probes of Kv1.3 channels on the membrane of eukaryotic cells. Recently, we have designed a plasmid that encoded the fluorescently labeled human Kv1.3 channel (mKate2-hKv1.3-del) with enhanced presentation on the membrane of eukaryotic cells [20]. This far-red fluorescent Kv1.3 channel was demonstrated to be able to bind specifically peptide blockers (hongotoxin 1 and its fluorescently labeled derivative) after transient expression in eukaryotic cells. Previously, the hKv1.3-del channels tagged with eGFP or mCherry were shown to be fully functional in the plasma membrane of mammalian cells [21]. 

**Figure 5:**
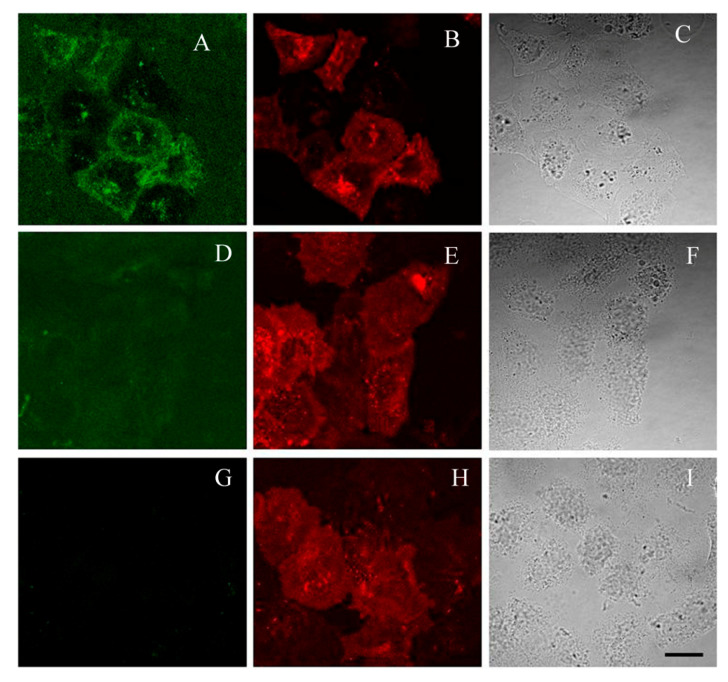
Confocal imaging of GFP-L2-AgTx2 interactions with mKate2-hKv1.3 channels, transiently expressed in HEK293 cells. (**A**–**C**) Cells that express mKate2-hKv1.3-del were stained with 40 nM of GFP-L2-AgTx2. (D-F) GFP-L2-AgTx2 (40 nM) was displaced from the complexes with mKate2-hKv1.3-del by AgTx2 (400 nM). (**G**–**I**) Cells that express mKate2-hKv1.3-del are shown without addition of GFP-L2-AgTx2. (**A**,**D**) Fluorescent images show distribution of GFP-L2-AgTx2. (**B**,**E**,**H**) Fluorescent images show distribution of mKate2-hKv1.3-del. The presented confocal fluorescent images were measured in the plane of a cell contact with a substrate, where a contribution of signals from the plasma membrane of cells dominates. (**C**,**F**,**I**) Transmitted light images of cells are shown. Bar corresponds to 20 μm.

Channel mKate2-hKv1.3-del was expressed in HEK293 cells (Figure 5G–I), and interactions of AgTx2-L2-GFP and His6-GFP-L2-AgTx2 with these cells were studied. It was found that incubation of cells with His6-GFP-L2-AgTx2 or GFP-L1-AgTx2 (40–100 nM for 30–60 min) resulted in staining of membranes of all cells both expressing and not expressing mKate2-hKv1.3-del (data not shown). Lower concentrations of His6-GFP-L2-AgTx2 or GFP-L1-AgTx2 (10 nM) did not demonstrate detectable membrane binding, while higher concentrations resulted in growth of bulk fluorescence interfering with the membrane signal. 

In contrast, after incubation with GFP-L2-AgTx2, membrane binding of the chimera was observed only in those cells, in which expression of mKate2-hKv1.3-del was high, and distribution of mKate2-hKv1.3-del at cell membrane correlated with that of GFP-L2-AgTx2 (Figure 5A–C). Specificity of this binding was demonstrated in the competitive binding experiment, when an excess of AgTx2 displaced totally GFP-L2-AgTx2 from the complexes with Kv1.3 at the cell membrane (Figure 5D–F). It should mentioned that our experiments did not reveal GFP-L2-AgTx2 binding to Kv1.1 channels on the membrane of HEK293 cells (not shown) that confirm selectivity of GFP-L2-AgTx2 to Kv1.3 channel. 

Results of these experiments indicate that GFP-L2-AgTx2 can be used as a fluorescent probe of Kv1.3 channels on membrane of eukaryotic cells, while His6-GFP-L2-AgTx2 and GFP-L1-AgTx2 are not suitable for this purpose because of non-specific membrane binding mediated, most probably, by His6-tag. 

### 2.6. Modeling of GFP-L2-AgTx2 and AgTx2-L3-GFP Chimeric Proteins

**Figure 6:**
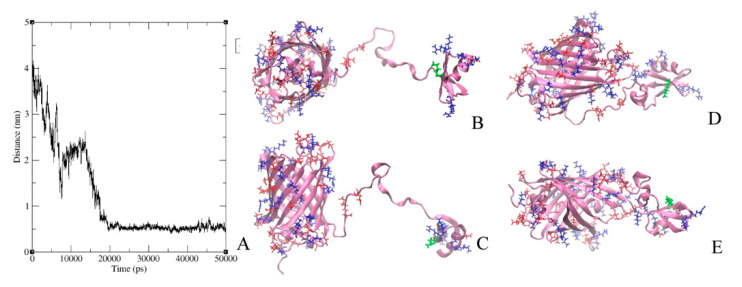
Formation of GFP-L2-AgTx2 intramolecular complex in molecular dynamics experiments. (**A**) Minimal distance between C_α_-atoms of GFP and AgTx2 during the 50 ns molecular dynamics simulation is shown. A stable intramolecular complex is formed after 20 ns. (**B**,**C**) A model of GFP-L2-AgTx2 is shown, where AgTx2 and GFP are initially situated far from each other (top (**B**) and side (**C**) views). (**D**,**E**) The same GFP-L2-AgTx2 model is presented after molecular dynamics simulation during 50 ns (top (**D**) and side (**E**) views). Acidic (Asp, Glu) and basic (Arg, Lys) residues are colored in red and blue, respectively. Lys27 of AgTx2, which interacts with a selective filter of channels, is shown in green.

**Figure 7:**
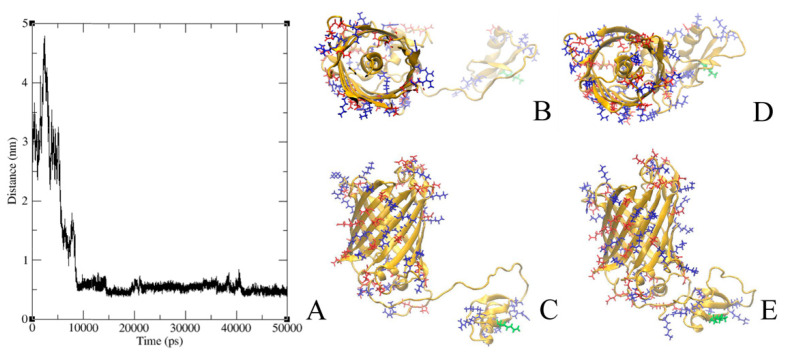
Formation of AgTx2-L3-GFP intramolecular complex in molecular dynamics experiments. (**A**) Minimal distance between C_α_-atoms of GFP and AgTx2 is shown during the 50 ns molecular dynamics simulation. A stable intramolecular complex is formed after 10 ns. (**B**,**C**) A model of AgTx2-L3-GFP is shown, where AgTx2 and GFP are initially situated far from each other (top (**B**) and side (**C**) views). (**D**,**E**) The same AgTx2-L3-GFP model is presented after molecular dynamics simulation during 50 ns (top (**D**) and side (**E**) views). Acidic (Asp, Glu) and basic (Arg, Lys) residues are colored in red and blue, respectively. Lys27 of AgTx2, which interacts with a selective filter of channels, is shown in green.

GFP-L2-AgTx2 and AgTx2-L3-GFP were characterized by molecular modeling methods aiming to evaluate their conformational dynamics in solution. For this, molecular dynamics simulations were performed for three models of GFP-L2-AgTx2 and three models of AgTx2-L3-GFP that differed in the initial mutual orientations of GFP and AgTx2 domains. In all the cases AgTx2 and GFP, which were initially situated far from each other (Figure 6B,C and Figure 7B,C), formed intramolecular complexes in 10–20 ns (only one representative model is presented for each chimeric protein), and these interactions were stable till the end of simulation (50 ns) (Figure 6A and Figure 7A). Interactions between AgTx2 and GFP were mediated by positively charged residues of AgTx2 and negatively charged residues of GFP (Figure 6D,E and Figure 7D,E) and numerous hydrogen bonds. Detailed patterns of these interactions depended on the initial conformation of a chimeric protein. In these experiments we verified and proved the possibility of direct intramolecular interactions between AgTx2 and GFP rather than tried to find the most stable conformation of GFP-L2-AgTx2 and AgTx2-L3-GFP. Evidently, interactions with GFP can prevent some AgTx2 residues of GFP-L2-AgTx2 and AgTx2-L3-GFP from interaction with potassium channels thus modulating free energy and probably the mode of AgTx2 binding to Kv1 channels. In contrast to GFP-L2-AgTx2 (Figure 6D,E), among final conformations of AgTx2-L3-GFP we observed the conformation, where pore blocking Lys27 as well as other residues, which are responsible for the interaction with Kv1 channels, were not screened by GFP (Figure 7D,E). 

## 3. Discussion

Peptide ligands of Kv1 channels fused with fluorescent proteins (FP-Tx) were demonstrated to preserve their high affinity binding to the outer vestibule of Kv1 channels, which was accompanied with blocking K^+^ currents through the channels [12]. Such FP-Tx are genetically encoded constructs and may represent an attractive alternative to radioactively and/or chemically labeled peptides in the studies of Kv1 channels and their blockers both in vitro and in vivo. Advantages of FP-Tx, which are produced in the *E. coli* expression system, are related to high levels of their biosynthesis, solubility and proper folding of FP-Tx in bacteria. Purification of FP-Tx by one- or two-step affinity procedure (Figure 1) does not require a renaturation step to get fluorescent peptide blockers in a functional form. The yields of purified GFP-OSK1 and RFP-AgTx2 were *ca*. 100 mg per 1 L of bacterial culture [12], while yields of GFP-L1-AgTx2, His6-GFP-L2-AgTx2, GFP-L2-AgTx2 and AgTx2-L3-GFP achieved 80, 100, 60 and 150 mg per 1 L of culture, respectively. 

Previous studies have demonstrated that tagging of OSK1 with GFP or tagging of AgTx2 with RFP did not considerably disturb the binding profiles of these peptides to eukaryotic Kv1.x (x = 1, 3, 6) channels [12]. Similarly, the FP-Tx ligands retained binding affinities of corresponding peptide blockers to the binding sites of Kv1.x (x = 1, 3, 6) channels that are presented in the hybrid proteins KcsA-Kv1.x (x = 1, 3, 6) [12]. In contrast, AgTx2 N-terminally tagged with GFP was found to lose essentially its ability to bind to Kv1.1 and Kv1.6 binding sites, but to preserve it for Kv1.3 (Figure 2). As a result, GFP-L2-AgTx2 reported in the present paper is an intriguing example of a genetically encoded selective fluorescent ligand of Kv1.3 channels, which are involved into pathogenesis of diabetes, psoriasis and some other diseases [22]. According to our estimation, N-terminal GFP enhances selectivity of AgTx2 to the Kv1.3 binding site by tenfold as compared to wild type AgTx2, and, finally, 200-fold selectivity is achieved for Kv1.3 over Kv1.1 and Kv1.6 binding sites. Such selectivity value appears to be sufficient for many applications that require a selective fluorescent ligand of Kv1.3 channel. 

The achieved selectivity is defined by GFP, when it is located at the N-terminus of AgTx2, because substitution of GFP with tagRFP or translocation of GFP to the C-terminus of AgTx2 results in the loss of this selectivity (Figure 4). Why does GFP but not tagRFP modulate the profile of FP-Tx interaction with the Kv1.x (x = 1, 3, 6) binding sites? The most obvious difference between GFP and tagRFP is a protein surface charge: −7 for GFP and −1 for tagRFP. Electrostatic interactions play a pivotal role in the binding of highly basic peptide toxins to negatively charged pore region of Kv1 channels, and negatively charged GFP can affect these interactions. While high structural and topological homology of the outer vestibule of the pore is inherent to Kv1 channels, a turret, the most protruding part of the outer binding site of the pore domain, comprises a stretch of anionic amino acid (a.a.) residues in each of four subunits, which varies among Kv1 channels: EEAE (Kv1.1), DDPT (Kv1.3), and DDDD (Kv1.6). A negatively charged array formed at the top of the channel pore is supposed to navigate a cationic peptide blocker during diffusion-limited stage of its binding to the channel, and some of these anionic residues are involved in the formation of complexes with AgTx2 [17,23]. Total net charge of the Kv1.3 binding site is less negative (−16) compared to the binding sites of Kv1.1 (−20) and Kv1.6 (−24) [17]. Evidently, a general effect of complex destabilization, which is associated with electrostatic repulsion of negatively charged GFP and the binding site of Kv1 channel, has to increase in the row Kv1.3 < Kv1.1 < Kv1.6. 

At the same time, the effect of electrostatic repulsion itself is not a critical factor explaining observed selectivity of GFP-L2-AgTx2, since previously studied GFP-OSK1 demonstrated high affinities to three channels Kv1.x (x = 1, 3, 6) [12]. It means that particular interactions of GFP with AgTx2, which abolish formation of bonds between specific amino acid residues of AgTx2 and Kv1.1 (Kv1.6), are responsible for the binding profile modulation when AgTx2 is a part of GFP-L2-AgTx2 ligand. The latter conclusion is consistent with the fact that transposition of GFP from N- to C-terminus of AgTx2 restores formation of complexes with the Kv1.1 and Kv1.6 binding sites, suggesting that changes in the spatial distribution of interacting a.a. residues of GFP, AgTx2 and a channel binding site determine the binding mode of GFP-L2-AgTx2 ligand. According to molecular modeling data an AgTx2-channel complex is stabilized by a complex network of bonds involving 10–11 channel residues and 16–18 peptide residues [17,23] that hamper prediction of the key interactions in AgTx2-channel complexes that are affected by GFP.

It should be mentioned that we did not find an effect of a linker connecting GFP with the N-terminus of AgTx2 on the selectivity of GFP-tagged AgTx2, when replaced the flexible short (22 a.a.) glycine-rich linker L2 with the flexible long (45 aa) linker L1. Molecular modeling studies of GFP-L2-AgTx2 revealed that GFP can form an intramolecular complex with AgTx2 even when a short linker is used (Figure 6). This result supports indirectly our hypothesis about GFP interference with specific bonds between some a.a. residues of AgTx2 and Kv1.1 (Kv1.6), which are important for stabilization of the AgTx2-channel complex. Different residues of AgTx2 are involved in the intramolecular interactions with GFP when GFP is translocated from N- to C- terminus of the peptide (Figure 6 and Figure 7). This difference presumably is responsible for modulating the effect of GFP on AgTx2 interactions with the binding sites of Kv1.x (x = 1, 3, 6) channels. 

It should be noted that the formation of intramolecular complexes predicted in the molecular modeling experiments for GFP-L2-AgTx2 and AgTx2-L3-GFP (Figure 6 and Figure 7) was demonstrated with X-ray crystallography for some chimeric proteins including GFP fused with ubiquitin binding motif of the mouse DNA polymerase iota (pdb-code 3AI4) [24] and circular permutated GFP fused with calmodulin (pdb-code 3EKJ) [25]. 

Our studies of GFP-L2-AgTx2, His6-GFP-L2-AgTx2 and GFP-L1-AgTx2 revealed that the presence of His6-tag, as well as its position within the FP-Tx molecule (N-terminal or as a part of L1 linker) does not affect the selectivity of a chimeric ligand, which is preserved in the 6.5–8.0 pH range. Comparison of properties of GFP-L2-AgTx2 and its structural precursor His6-GFP-L2-AgTx2 shows that any of these ligands can be efficiently used as a selective fluorescent probe in the bioengineering analytical system on the basis of KcsA-Kv1.3 channel to search for Kv1.3 channel blockers (both among individual compounds and in complex mixtures) and to estimate their activity (Figure 3E,F, Table 1). In this application, His6-GFP-L2-AgTx2 is more advantageous than GFP-L2-AgTx2 because (i) it can be produced and purified in one stage with high yield (Figure 1), and (ii) it is slightly more active (Figure 3C,D). The elevated activity of His6-GFP-L2-AgTx2 can be presumably related to the presence of slightly positively charged His6-tag (pI = 7.75) that partially neutralize a negative charge of GFP. High affinity of GFP-L2-AgTx2 and His6-GFP-L2-AgTx2 to the Kv1.3 binding site was revealed in the pH range of 6.5–7.5 (Figure 3C,D). Decrease in *K_d_* at pH 8.0 can be explained by deprotonation of some cationic residues of GFP-tagged AgTx2, in particular, related to AgTx2. AgTx2 is a very basic peptide (pI = 9.0), and its net charge changes from 6.4 at pH 6.5 to 4.0 at pH 8.0, while the net charge of the P-loop of the Kv1.3 binding site (pI = 3.25) is highly negative at these pH values.

As demonstrated, GFP-L2-AgTx2 can be also applied for investigation of Kv1.3 channels at the membrane of eukaryotic cells, where it produces specific complexes with the channels (Figure 5). In contrast, His6-GFP-L2-AgTx2 and GFP-L1-AgTx2 are not suitable for these purposes, since, as noted above, His6-tag enhances non-specific membrane interactions, which mask formation of the specific complexes. The basis of non-specific binding may be accounted for a slight positive charge of poly-histidine at pH values near 7.0, which can be even increased by the local changes in the pK of the imidazole group induced by the neighboring residues. Thus, overhanging His6-tag is able to interact with anionic membrane molecules enhancing non-specific binding. Similar effect of intermolecular interactions of protonated His6-tag with anionic protein areas at slightly acidic pH was proposed for self-assembly of nanoparticles [26]. Additionally, the density of channels on the membrane of HEK293 cells is much less than on the membrane of spheroplasts, while the area of the membrane itself is much larger that apparently, determines an increase in the contribution of non-specific binding of His6-GFP-L2-AgTx2 and GFP-L1-AgTx in experiments with eukaryotic cells.

To our best knowledge, GFP-tagged AgTx2 chimeras are the first examples of ionic channel ligands, where a high selectivity to a particular channel was achieved due to tagging the ligand with a fluorescent protein instead of a conventional search for a.a. substitutions that modulate the binding profile of a ligand. 

## 4. Conclusions

On the basis of our new and previously obtained results we conclude that by combining different peptides blockers with various fluorescent proteins the genetically encoded fluorescent FP-Tx ligands of Kv1 channels can be created, which have high affinity to the target channels, fluoresce in different spectral regions and possess potential both for in vitro and in vivo applications. Moreover, particular combinations of fluorescent proteins and peptide blockers can result in considerable modulation of the initial blocker-channel binding profile yielding the selective fluorescent ligands of particular channels. Currently, a rational prediction of such combination is hardly possible, and the experimental search for FP-Tx with unusual properties is required.

## 5. Materials and Methods 

### 5.1. Gene Design, Synthesis and Cloning

Genes coding for GFP-L1-AgTx2, His6-GFP-L2-AgTx2, and MBP-L1-AgTx2-L3-GFP were constructed and cloned into pET-23d expression vector (Novagen) to obtain corresponding chimeric proteins. The GFP-L2-AgTx2 chimera was obtained by hydrolysis of His6-GFP-L2-AgTx2 with TEV protease. AgTx2-L3-GFP was produced by TEV hydrolysis of MBP-L1-AgTx2-L3-GFP. Schematic representation of expression cassettes and chimeric proteins is given in Figure 1. Oligonucleotide primers used for cloning are shown in Table 2. The structures of linkers are as follows: 

L1 (45 aa)—KNSGSGSGHMHHHHHHSSGLVPRGSGMKETAAAKFERQHMDSPGT;

L2 (22 aa)—EGGSGGSGGTGGAGGAGGTGGS;

L3 (20 aa)—GSGGSGGSGGTGGAGGATST. 

To construct pET23-GFP-L1-AgTx2 (Figure 1A), a gene coding for RFP in the previously obtained plasmid pET23-RFP-L1-AgTx2 [12] was replaced by the GFP coding sequence using NcoI/EcoRI sites. For this, the GFP gene was PCR-amplified from pUC-eGFP plasmid using oligonucleotide primers GFP-f1 and GFP-r1. 

To obtain plasmid pET23-His6-GFP-L2-AgTx2, GFP gene was produced in two rounds of PCR. First, pUC-GFP plasmid, and primers GFP-f2 and GFP-r2 were used to amplify a 760-bp DNA fragment I to get the GFP gene elongated at its 5’- and 3’-termini. In the second round of PCR, fragment I was further elongated from its 5’-terminus using GFP-f3 and GFP-r2 primers to obtain the 796-bp fragment II. AgTx2 gene was elongated at its 5’-terminus and PCR-amplified using oligonucleotides Ag-1f, Ag-2f, Ag-1r, and Ag-2r to get DNA fragment III. Fragments II and III were digested using restriction enzymes NcoI/KpnI and KpnI/HindIII, respectively, and cloned into NcoI/HindIII sites of pET-23d vector (Novagen). Resulting vector pET23-His6-GFP-L2-AgTx2 coded for chimeric His6-GFP-L2-AgTx2 protein, in which GFP and AgTx2 moieties were separated by L2 linker (Figure 1B). N-terminal extension of GFP comprised His6 tag, as well as TEV protease cleavage site (CS_TEV_, ENLYFQG), which was further used to produce GFP-L2-AgTx2 (Figure 1B). BamHI cleavage site was introduced before AgTx2 gene so that the expression cassette can be used for cloning genes other than AgTx2. 

Cloning of a gene coding for C-terminally GFP-tagged AgTx2, namely AgTx2-L3-GFP, was carried out using previously obtained pET23-MalE-L1-AgTx2 as an expression vector [17]. First, GFP gene was PCR-amplified using primers GFP-f4 and GFP-r3 to get DNA fragment IV, which was further amplified in the second round of PCR using primers GFP-f5 and GFP-r3 to get DNA fragment V. SphI restriction cleavage site located within the AgTx2 gene of the vector, and XhoI unique restriction site from the multiple cloning site of 

pET23d vector were used to clone SphI/XhoI-digested fragment V into pET23-MalE -L1-AgTx2 to obtain pET23- MalE-L1-AgTx2-L3-GFP (Figure 1C). Stop TAA codon was introduced into GFP-r3 primer immediately before XhoI site, thus, the only hexahistidine tag in the chimeric protein MBP-L1-AgTx2-L3-GFP was located within the L1 linker. For further cloning, restriction sites SalI and BamHI were introduced into GFP-f4 and GFP-f5 primers, respectively. Protein MBP-L1-AgTx2-L3-GFP was used to produce AgTx2-L3-GFP by TEV protease digestion (Figure 1C). The synthesis of oligonucleotide primers and the DNA sequencing of constructed plasmids were performed by Evrogen (Russia). Correct cloning of target genes was confirmed by sequencing of both strands.

### 5.2. Expression and Purification of GFP-Tagged AgTx2 

Chimeric proteins were expressed as described earlier [12]. Briefly, *E. coli* Rosetta-gami(DE3)pLysS cells transformed with a plasmid (pET23-GFP-L1-AgTx2, pET23-His6-GFP-L2-AgTx2, or pET23- MalE-L1-AgTx2-L3-GFP) were cultivated at 37 °C in 0.1 L of Terrific Broth (TB) medium in the presence of 100 mg/L ampicillin, 15 mg/L kanamycin, 12.5 mg/L tetracycline, and 34 mg/L chloramphenicol to the mid-log phase, then induced with 0.1 mM isopropyl β-D-thiogalactopyranoside (IPTG) and further incubated at 20 °C for 22 h. Cells were harvested by centrifugation, disrupted by sonication, and chimeric GFP-tagged AgTx2 proteins were purified from the soluble fraction by affinity chromatography on a Ni-NTA Sepharose Fast Flow column (1.5 mL bed volume, GE Healthcare) following the manufacturer’s protocol. The proteins were desalted using PD-10 columns (GE Healthcare) in PBS (pH 7.4), and stored at +4 °C in the dark in the presence of 0.02% sodium azide. Denaturing SDS-PAGE was used to analyze the level of recombinant protein biosynthesis, as well as to characterize the purified FP-Tx (Figure 1D–F). Concentrations of chimeric proteins in solution were determined by absorption spectroscopy using molar extinction coefficient for GFP (ε_489_ = 55,000 M^−1^ cm^−1^). The yields of GFP-L1-AgTx2, His6-GFP-L2-AgTx2 and MBP-L1-AgTx2-L3-GFP were 80, 100 and 250 mg per 1 L of culture, respectively.

### 5.3. Preparation of GFP-L2-AgTx2 and AgTx2-L3-GFP 

Chimeric proteins, His6-GFP-L2-AgTx2 and MBP-L1-AgTx2-L3-GFP, obtained as described above were subjected to TEV protease hydrolysis according to [27]. Before hydrolysis, the PBS buffer was exchanged for buffer F (50 mM Tris-HCl, pH 8.0, 100 mM NaCl, 1 mM EDTA) by protein desalting on PD-10 columns. 

Reaction mixture containing chimeric protein (2 g/L), TEV protease (0.4 g/L), and 0.5 mM dithiothreitol in buffer F was incubated at 20 °C for 4 h. Cleavage products were subjected to affinity chromatography using Ni-NTA Sepharose Fast Flow resin to remove His6-tagged proteins including His6-TEV protease. Each of the purified chimeric protein, GFP-L2-AgTx2 or AgTx2-L3-GFP, was collected from the flow-through, desalted on a PD-10 column in PBS (pH 7.4), concentrated on an Amicon Ultra 10 kDa filters (Millipore) to 1.5 mg/mL. The obtained proteins were characterized with SDS-PAGE (Figure 1D–F) and stored as described above. The yields of GFP-L2-AgTx2 and AgTx2-L3-GFP achieved 60 and 150 mg per 1 L of culture, respectively.

### 5.4. Recombinant Toxins

Recombinant peptides, namely ChTx, KTx1, AgTx2, HeTx, and ScTx were obtained as described earlier [27,28].

### 5.5. Venoms

Venoms of scorpions *Mesobuthus eupeus* and *Orthochirus scrobiculosus* were kindly gifted by Dr. A. Vassilevski. 

### 5.6. Preparation of Spheroplasts and Binding Protocol

*E. coli* cells expressing KcsA-Kv1.x (x = 1, 3, 6) were cultured as described elsewhere [15,16,17]. Spheroplasts were prepared according to the procedure described earlier [11]. For binding experiments, spheroplasts prepared from non-transformed *E. coli* cells, spheroplasts expressing KcsA, KcsA-Kv1.1, KcsA-Kv1.3 or KcsA-Kv1.6 were incubated (1000 cells μL^−1^, 2 h, 37 °C) with GFP-L2-AgTx2 (40 nM), His6-GFP-L2-AgTx2 (40 nM), GFP-L1-AgTx2 (40 nM) or AgTx2-L3-GFP (20 nM) in buffer A containing 0.1% bovine serum albumin, 250 mM sucrose, 10 mM MgCl_2_, 10 mM Tris-HCl (pH 7.5), 4 mM KCl and 0.3 mM EDTA. Concentration dependencies of KcsA-Kv1.3 binding to GFP-tagged AgTx2 were measured varying concentrations of GFP-L2-AgTx2 (0.625–50 nM) or His6-GFP-L2-AgTx2 (0.625–40 nM) in buffer A at different pH (2 h incubation at 37 °C). Competitive binding was assessed by incubating KcsA-Kv1.3-presenting spheroplasts with GFP-tagged AgTx2 (20 nM) and different concentrations of non-labeled peptide ligands (AgTx2, ChTx, HeTx, KTx1 or ScTx) or crude scorpion venom for 2 h at 37 °C. In the detailed competitive binding experiments (Figure 3E,F) concentrations of AgTx2, ChTx, HeTx and KTx1 varied in the range of 0.05–6.3 nM, 0.8–200 nM, 2–2000 nM and 0.01–5 nM, respectively. 

### 5.7. Construction of mKate2-KCNA3-del Gene

KCNA3 gene encoding human Kv1.3 (hKv1.3, Accession number NP_002223.3) was a generous gift from Dr. A. Vassilevski. The gene was amplified using primers KCNA3-del-f1 and KCNA3-r1 (Table 2) to obtain KCNA3-del gene encoding N-terminally truncated protein, in which residues M1 through D52 were deleted [29]. The truncated gene was inserted into BglII/HindIII sites of pmKate2-C plasmid (Evrogen, Moscow, Russia) to obtain the resulting plasmid pmKate2-KCNA3-del, which was further used for transfection of HEK293T cells. In the hybrid protein, mKate2-hKv1.3-del, mKate2 was linked to the N-terminus of the hKv1.3-del channel through the 9-aa flexible linker. Correct cloning of the target gene was confirmed by sequencing of both strands in the created plasmid (Evrogen, Russia).

### 5.8. Experiments with Cells 

HEK 293 cells (from the collection of the Institute of Cytology RAS) were grown (37 °C, 5% CO_2_) in DMEM/F12 (Paneco, Russia) containing 5% fetal bovine serum (FBS, HyClone, Utah, USA) and 2 mM L-glutamine. Cells were passaged every 72 h. Cells used in the experiments were from the 5–20 passages. Transient transfection of cells with the plasmid encoding mKate2-hKv1.3-del was performed using Unifectin 56 (UnifectGroup, Russia) at nearly 50% confluence according to the manufacturer’s protocol. Twenty four hours after transfection, cells were rinsed twice with a buffer (20 mM Hepes pH 7.4, 260 mM sorbite, 4 mM KCl, 10 mM NaCl, 1.8 mM CaCl_2_, 1 mM MgCl_2_, 0.1% bovine serum albumin) and incubated in this buffer with GFP-L2-AgTx2 (40 nM) for 40 min. Competitive binding was assessed by incubating cells with GFP-L2-AgTx2 (40 nM) and AgTx2 (400 nM) for 40 min. Similarly, His6-GFP-L2-AgTx2 and GFP-L1-AgTx2 were incubated with cells at 10–100 nM concentrations for 30–60 min. 

### 5.9. Microscopy 

Experiments with *E. coli* spheroplasts were carried out with a laser scanning confocal microscope LSM 710 (Zeiss, Germany). An α Plan-Apochromat 100×/1.46 oil immersion objective was used. Fluorescence of GFP-tagged AgTx2 was excited at the 488 nm wavelength and recorded in the 495–590 nm range at 0.25 μm lateral and *ca.* 1.5 μm axial resolution. To accomplish quantitative analysis of the binding of GFP-tagged AgTx2 to spheroplasts, all the parameters of measurements that have an effect on intensity of the recorded signal were fixed. The analysis was performed as reported earlier [15,16,17]. Briefly, the recorded fluorescent images were treated with the ImageJ software (National Institutes of Health, Bethesda, MD, USA) to recognize fluorescent spheroplasts and calculate the average fluorescence intensity of GFP-tagged AgTx2 associated with each cell (*I_f_*). *I_f_* values of 140–240 spheroplasts were averaged to obtain the *I_av_* value (±SEM). 

Dissociation constants (*K_d_*) of complexes were calculated from the measured *I_av_* dependencies on the concentration of GFP-tagged AgTx2 using equation:

*I_av_* ([L]) = *I_sat_* [L]/(*K_d_* + [L]), (1)

where [L] is a concentration of GFP-tagged AgTx2, *I_sa_*_t_ is the *I_av_* value at saturated binding.

Apparent dissociation constants (*K_ap_*) of non-labeled Kv1 channel pore blockers were defined from the competition binding experiments as published earlier [15,16,17] using the Cheng-Prusoff equation:

*K_ap_* = *IC_50_*/(1 + [*L*]/[*K_d_*]), (2)

where [*L*] is a concentration of GFP-tagged AgTx2 (20 nM in our experiments), *IC_50_* is the concentration of a competing peptide displacing 50% of GFP-tagged AgTx2 from the complex with KcsA-Kv1.x (x = 1, 3, 6). 

The *K_d_* and *K_ap_* values were averaged over three independent experiments and presented as mean ± SEM.

Experiments with HEK293 cells were performed with a laser scanning confocal microscope Leica-SP2 (Leica Microsystems GmbH, Wetzlar, Germany). The water-immersion 63×/1.2 NA HCX PL APO objective was used. Fluorescence of GFP-tagged AgTx2 was excited at the 488 nm wavelength and recorded in the 495–535 nm range. Fluorescence of mKate2-hKv1.3 was excited at the 561 nm wavelength and recorded in the 650–700 nm range.

### 5.10. Optical Spectroscopy 

**Figure 8:**
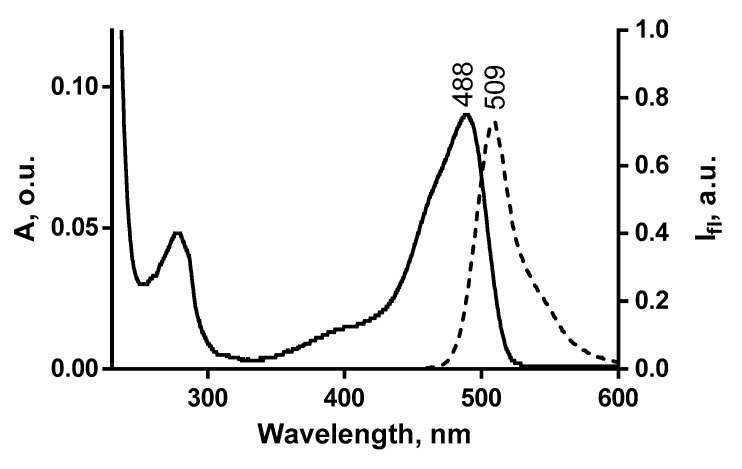
Absorption (solid line) and fluorescence (dashed line) spectra of GFP-L2-AgTx2 in phosphate-buffered saline.

Absorption and fluorescence spectra were measured for GFP-tagged AgTx2 in phosphate-buffered saline using Cary 50 spectrophotometer and Cary Eclipse spectrofluorimeter (Varian, Inc., Paolo Alto, CA, USA). Absorption and fluorescence spectra of studied GFP-tagged AgTx2 were found to be very similar in a visible region, and they are typical for GFP. An example of absorption and fluorescence spectra of GFP-tagged AgTx2 is shown for GFP-L2-AgTx2 in Figure 8. 

### 5.11. Computer Modeling of Chimeric Proteins GFP-L2-AgTx2 and AgTx2-L3-GFP

Initial structural models of hybrid proteins GFP-L2-AgTx2 and AgTx2-L3-GFP were obtained by homology modeling based on the structures of GFP (pdb-code 2Y0G) and AgTx2 (pdb-code 1AGT). These structures were placed in three different mutual orientations and then used as a template for Modeler 9.14. Linkers were mainly in disordered conformation. GFP chromophore was replaced with the sequence of TYG which is similar in size and does not complicate protein topology preparation. Then initial models were prepared for the simulation of molecular dynamics with the use of Gromacs 2020 [30] and CHARMM36 force field [31]. Typical box was 10 × 10 × 10 nm by size and contained 10^5^ atoms including protein, water molecules and ions. Trajectories with the length of 50 ns were obtained and then visually inspected with VMD 2.3.1 [32]. Interactions aroused between GFP and AgTx2 moieties were analyzed using Gromacs built-in tools.

## Figures and Tables

**Table 1:** Apparent dissociation constants *K_ap_* of complexes between different peptide blockers and the Kv1.3-binding site calculated on the basis of competitive binding experiments performed with either GFP-L2-AgTx2 or His6-GFP-L2-AgTx2 fluorescent ligand.

	*K_ap_* (pM)			
Fluorescent Ligand	AgTx2	KTx1	ChTx	HeTx
GFP-L2-AgTx2	230 ± 50	100 ± 40	1500 ± 300	39,000 ± 4000
His6-GFP-L2-AgTx2	70 ± 4	71 ± 7	900 ± 170	45,000 ± 8000

**Table 2:** Primers used in this study.

Notation	Nucleotide Sequence *
GFP-f1	5′-TTCTTCCATGGTGAGCAAGGGCGAGGAGCTGTT-3′
GFP-r1	5′-TTCTTGAATTCTTGTACAGCTCGTCCATGCCGAGA-3′
GFP-f2	5′-CACAGCAGTGGCGAAAACCTGTACTTTCAGGGTATGGTGAG CAAGGGCGAG-3′
GFP-r2	5′-TTCTT**GGTACCTCCCGAACCTCCCGAGCCTCCCTC** GTAC AGCTCGTCCATGCCGA-3′
GFP-f3	5′-CCTCCTCCATGGGCAGCTCTCATCACCATCACCATCACAGCAGT GGCGAAAAC-3′
Ag-1f	5′-TTCTA**GGTACCGGTGGCGCGGGAGGCGCGGGAGGTACGG** **GTGG*ATCC*** *GGCGTTCCGA*-3′
Ag-2f	5′-GGTTCTCCACAGTGTATCAAACCGTGCAAAGATGCAGGCA*TGC* *GCTTTGGCAAATG*-3′
Ag-1r	5′-TGATACACTGTGGAGAACCCGTGCAGCTCACGTTGA*TCGG* *AACGCCGGAT*-3′
Ag-2r	5′-TTCTTCAAGCTTACTTCGGCGTGCAGTGACACTTACGATT CATG*CATTTGCCAAAGCGCA*-3′
GFP-f4	5′-**CGGAGGTAGTGGAGGCAGCGGAGGCACCGGTGGCGCAG** **GCGGTGCAACGTCGACC**ATGGTGAGCAAGGGCGAG-3′
GFP-r3	5′-TTCTTCTCGAGTTACTTGTACAGCTCGTCCATGCCGAGAG-3′
GFP-f5	5′-TGCAGGCATGCGCTTTGGCAAATGCATGAATCGTAAGTGTC ACTACGCCGAAG**GGATCCGGAGGTAGTGGAGGCA**-3′
KCNA-del-f1	5′-TTCTCAGATCTATGACCGTGGTGCCCGGGGACCA-3′
KCNA-r1	5′-TTCTCAAGCTTAAACATCGGTGAATATCTTTTTGATGTTGA-3′

* Restriction enzyme sites used for cloning are underlined. Restriction endonuclease cleavage sites introduced in Ag-1f and GFP-f5 primers (BamHI) and in GFP-f4 primer (SalI) are also underlined. Overlapping sequences are shown in italics. Nucleotide sequences coding for L2 linker (in primers GFP-r2 and Ag-1f) and for L3 linker (in primers GFP-f4 and GFP-f5) are marked in bold. Coding sequences for TEV cleavage site in GFP-f2 primer and for His6 tag in GFP-f3 primer are underlined. Start (ATG) codons for GFP gene in primers GFP-f1, GFP-f2, and GFP-f4, as well as for KCNA3-del gene in primer KCNA3-del-f1 are shadowed gray.
